# Congenital Langerhans Cell Histiocytosis With the Skin and Lung Involvement: A Case and Literature Review

**DOI:** 10.7759/cureus.49453

**Published:** 2023-11-26

**Authors:** Romane Teshima, Yumiko Sakuragi, Natsuko Saito-Sasaki, Etsuko Okada, Yu Sawada

**Affiliations:** 1 Dermatology, Hospital of University of Occupational and Environmental Health, Kitakyushu, JPN; 2 Dermatology, Hospital of University of Occupational and Environmental Health, Kitakyusyu, JPN

**Keywords:** lung and skin lesions, chemotherapy, congenital, literature review, langerhans cell histiocytosis(lch)

## Abstract

Langerhans cell histiocytosis (LCH) is a clonal proliferative disease of immature Langerhans cells that expand in various organs, leading to organ and tissue dysfunction. Although LCH is most commonly seen in children under the age of three, a small number of cases of congenital LCH have been described. With a review of the literature on congenital LCH with lung and skin lesions, we present a case of congenital LCH with involvement of skin and lung, which was effectively treated with chemotherapy without recurrence for 3 years during the observational period. In addition, we summarized previously published case studies of congenital LCH with skin and lung involvement.

## Introduction

Langerhans cell histiocytosis is a disease in which clonal Langerhans cells bear the immature dendritic cell characteristics derived from the bone marrow, and the tumor cells aggregate in the various organs, such as bones, skin, and central nervous system, leading to tissue destruction [1]. A recent updated study showed that the most common site of occurrence is bones, especially the skull, and skin lesions are also seen in about 77.7% of cases recognized as the first symptoms of LCH [2]. Although LCH is mostly observed in the pediatrics period under the age of three, a limited number of cases with congenital LCH have been reported [3] [4] [5] [6] [7]. In addition, the multi-system type of LCH was recognized as an unfavorable clinical course. Although some cases of congenital LCH showed a spontaneous regression, the clinical outcomes of multi-system types of congenital LCH remain unclear. Herein, we report a case of congenital multi-system type of LCH involved in the skin and lung, which was successfully treated by chemotherapy without recurrence. In addition, we summarized a review of the literature on congenital LCH with lung and skin lesions to determine the clinical characteristics and the outcomes of these cases.

## Case presentation

**Figure 1:**
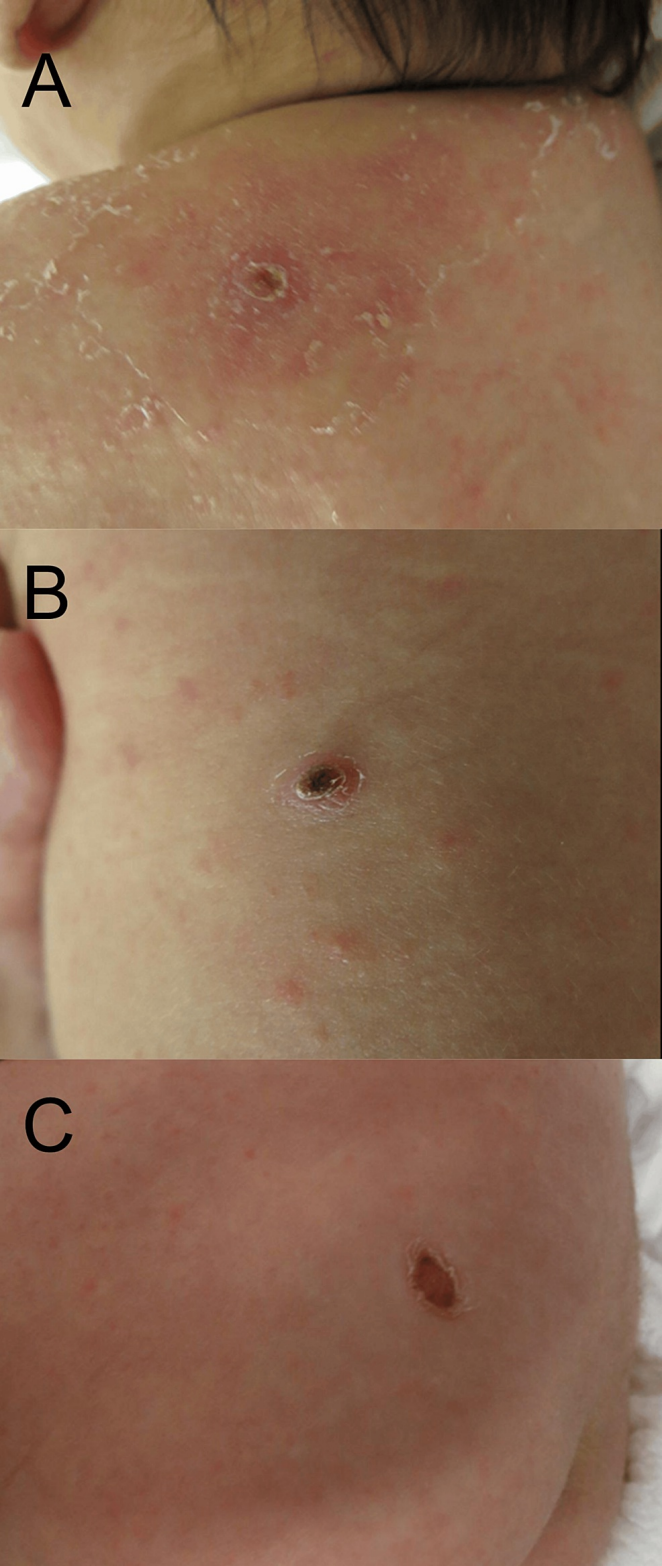
Clinical manifestations. A physical examination showed that solid papules with crusts were observed on the left shoulder (A), left back (B), left buttock (C) on the 31st day of the birth.

**Figure 2:**
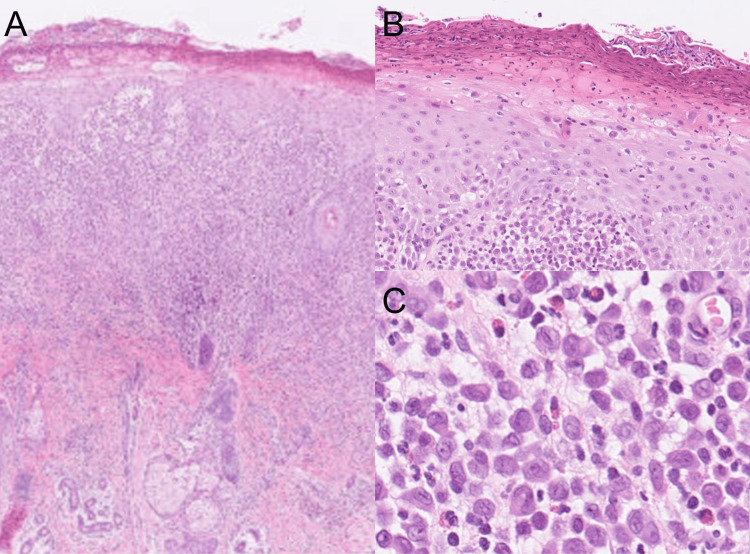
Histological examinations. A skin biopsy taken from her skin eruption showed hyperkeratosis and parakeratosis with a mild spongiosis in the epidermis (A: A low magnification view) (B: A high magnification view). Histiocytoid cells with coffee bean-shaped irregular nuclei proliferated from the upper to middle dermal layers, and were mixed with infiltration of lymphocytes and eosinophils (Figure 2C).

**Figure 3:**
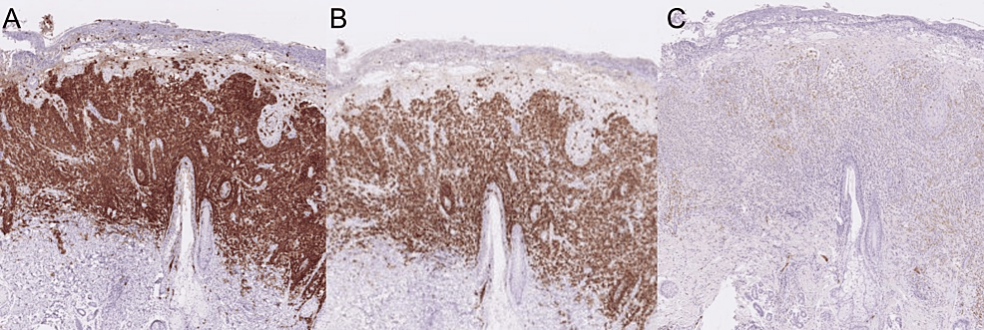
Immunohistochemical staining examination Immunohistochemical staining examination showed positive reactions to (A) CD1a, (B) Langerin, and (C) S-100.

A newborn female exhibited erythematous plaques and multiple 2 to 3-mm blisters on her lower trunk and extremities at birth, which quickly altered into crusts after birth. Since her skin eruption was intractable by the topical corticosteroid ointment treatment, she was referred to our department to evaluate her skin eruption on the 31st day of the birth. A physical examination showed that solid papules with crusts were observed on the left shoulder, left back, and left buttock (Figure 1A-1C). No fungi were detected by KOH microscopy examination. A skin biopsy taken from her solid papules on the shoulder showed hyperkeratosis and parakeratosis with a mild spongiosis in the epidermis (Figures 2A, 2B). Histiocytoid cells with coffee bean-shaped irregular nuclei proliferated from the upper to middle dermal layers and were mixed with infiltration of lymphocytes and eosinophils (Figure 2C). Immunohistochemical staining examination revealed that the tumor cells were positive for CD1a, Langerin, and S-100 (Figure 3A-3C).

**Figure 4:**
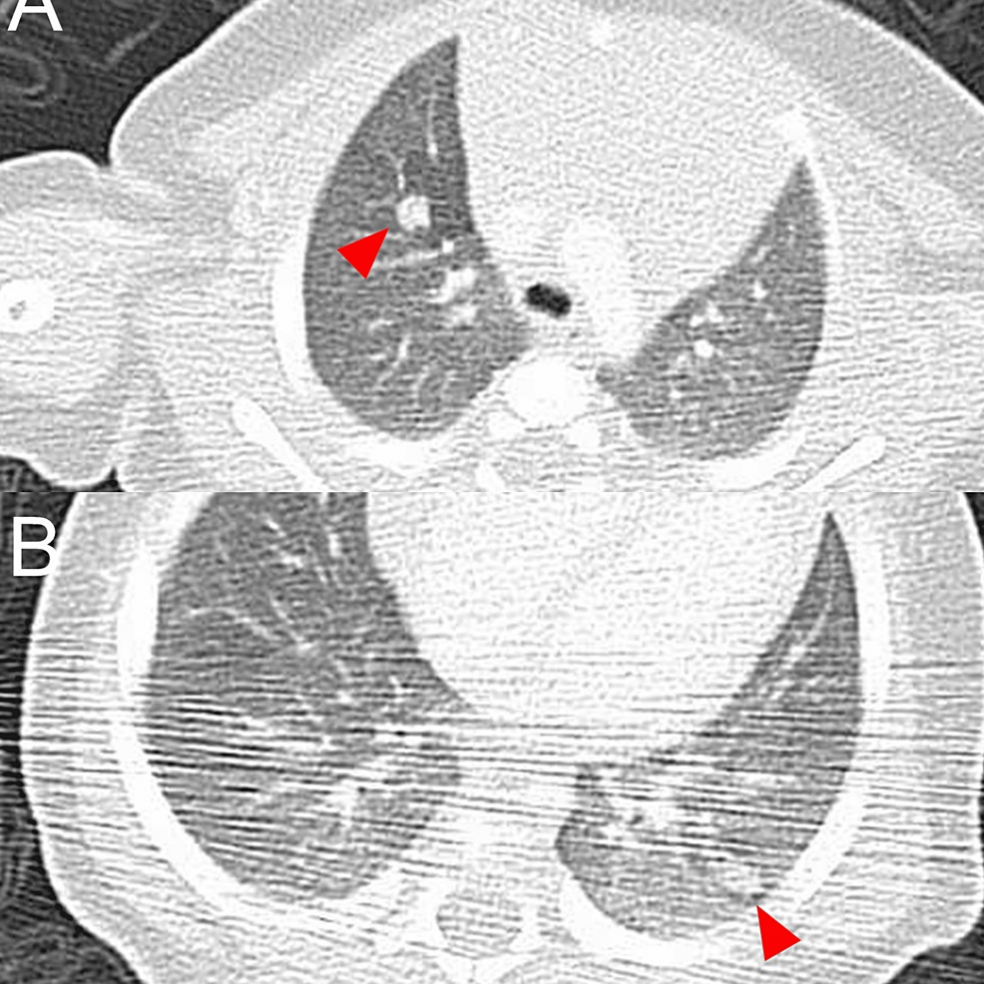
Computed tomography examination A chest computed tomography imaging revealed clearly demarcated multiple nodules in both lung (A and B). Red arrow heads indicate the nodules in the lung.

A chest computed tomography imaging revealed clearly demarcated multiple nodules in both lung (Figure 4A, 4B). CT of the head, abdomen, and pelvis revealed no obvious abnormal findings. No obvious bone lesions were found on X-ray examinations.

Based on these findings, this patient was diagnosed with LCH in the skin and lung lesions, considered a multiple organ type. Therefore, the chemotherapy based on the protocol of JLSG-02 was administered. This patient was treated with six weeks of induction A chemotherapy consisting of Cytarabine (Ara-C)/ Vincristine sulfate (VCR)/ Prednisolone (PSL) and subsequently received Early Maintenance A (Ara-C/VCR,/PSL and Methotrexate (MTX)/VCR/PSL in 6 times each alternately) and Late Maintenance C therapies (Vinblastine (VBL)/PSL/6-MP and MTX/6-MP in 6 times each alternatively). The skin and lung lesions rapidly disappeared, and the patient remained in remission for three years after the treatment.

## Discussion

The multi-system type of LCH exhibits an unfavorable clinical behavior and requires systemic chemotherapy even in patients without the risk of organ involvement, such as the liver, spleen, and bone marrow [8,9]. In addition, our case also exhibited unfavorable clinical factors, namely, being younger than 2 years old and having high serum sIL-2R levels [10,11]. Because there was a limited number of cases with congenital LCH in both skin and lung, the clinical behaviors and outcomes remain unclear.

To clarify this issue, we reviewed English- and Japanese-reported cases of LCH with skin and long involvement and summarized them in Table 1 [3-7]. In total, 7 cases have been reported, including our patients. The male-to-female ratio in these studies is 4:3. Several types of skin eruptions exist. The most common types of skin eruptions were nodules or papules (5 cases), followed by ulceration or erosion with crust (2 cases). Several cases exhibited other systemic organ involvement, such as liver (2 cases), bone (2 cases), and spleen (1 case). Two cases received chemotherapy against LCH and showed complete remission in all cases.

**Table 1:** A summary of previously published cases of congenital Langerhans cell histiocytosis with the skin and lung lesions

Authors	Sex	Skin eruption	Other lesion	Treatment	Prognosis	Observation period
Aggarwal V, et al. [3]	Male	Nodule and hypopigmented macules	None	Observation	Survival	4 months
Stiakaki E, et al. [4]	Female	Nodules	None	mPSL 1mg/kg	Survival	20 months
	Male	Nodules	None	mPSL 1mg/kg	Survival	5 months
Tamefusa K, et al. [5]	Male	Ulcer with crust and abscess	Liver, Spleen, Skull, Thymus gland	Chemotherapy	Survival	4 years
Nakashima T, et al. [6]	Female	Erosion vesicles	None	Observation	Survival	2 years
Mandel VD, et al. [7]	Male	Papules	Liver, Skull	Observation	Survival	30 months
Our case	Female	Nodules and papules with crust	None	Chemotherapy	Survival	3 years

On the contrary, three cases were kept under observation, and all cases showed complete remission. The other two cases received methylprednisolone 1mg/kg, and these cases also showed complete remission. Although all these cases exhibited favorable clinical behavior without recurrence of skin eruption by the observation, further investigation is required to conclude the actual clinical course and therapeutic outcomes in LHC with skin and lung involvement.

## Conclusions

We experienced a rare case of congenital LCH in skin and lung involvement. Given that only seven relative cases have been published, further case studies should be available to investigate the clinical characteristics of these cases. The actual clinical outcomes are required to be concluded by further accumulation of case studies.
